# Retracted: Electroadsorption Desalination with Carbon Nanotube/PAN-Based Carbon Fiber Felt Composites as Electrodes

**DOI:** 10.1155/2016/8469373

**Published:** 2016-07-25

**Authors:** The Scientific World Journal

The Scientific World Journal has retracted the article titled “Electroadsorption Desalination with Carbon Nanotube/PAN-Based Carbon Fiber Felt Composites as Electrodes” [1]. The article was found to contain figures repurposed, without citation, from previously published articles.
